# Advances in ITP – Therapy and Quality of Life – A Patient Survey

**DOI:** 10.1371/journal.pone.0027350

**Published:** 2011-11-11

**Authors:** Axel C. Matzdorff, Gabriele Arnold, Abdulgabar Salama, Helmut Ostermann, Sonja Eberle, Simone Hummler

**Affiliations:** 1 Department of Haematology, Caritasklinik St. Theresia, Saarbruecken, Germany; 2 ITP Self-Support Group, Giessen, Germany; 3 Institute for Transfusion Medicine, Charité -Universitaetsmedizin Berlin, Berlin, Germany; 4 Department of Haematology, University of Munich - Grosshadern Campus, Munich, Germany; 5 Department of Biostatistics and Epidemiology, GlaxoSmithKline, Munich, Germany; 6 Division of Preventive Oncology, National Center for Tumor Diseases and German Cancer Research Center, Heidelberg, Germany; Oklahoma Medical Research Foundation, United States of America

## Abstract

**Background:**

Current guidelines recommend glucocorticoids and splenectomy as standard 1^st^ and 2^nd^ line treatments for chronic immune thrombocytopenia (ITP). We sought to find out how German ITP-patients are treated with respect to these guidelines.

**Methods:**

Members of a patient support association ≥18 years with a self-reported history of chronic ITP>12 months were surveyed with a web-based questionnaire.

**Results:**

122 questionnaires were evaluated. 70% of patients had chronic ITP for more than 5 years and 20% an average platelet count of ≤30·10^9^/L. 41% of the patients reported haematomas or petechiae more than once or twice and up to 12 times or more per year and 17% oropharyngeal and nasal bleeds. 11% had been admitted to hospital during the last 12 months. 88% had received or currently receive glucocorticoids, 27% were splenectomised. IVIG had been given to 55%, rituximab to 22%, anti-D to 12%, ciclosporin to 7%, while complementary and alternative medical treatments had been used by 36%. 50 women responded to questions concerning pregnancy. 14 (28%) had been advised not to become pregnant. 23 reported pregnancies and 10 (44%) required treatment for their ITP during pregnancy.

**Conclusion:**

Glucocorticoids are the most common therapy for chronic ITP but complementary and alternative treatments already come second and less than ⅓ of patients are splenectomised. This and the frequent use of complementary medicines suggests patients' dissatisfaction with conventional approaches. Many patients receive off-label therapies. There is a major need for adequate counselling and care for pregnant ITP-patients.

## Introduction

Chronic immune thrombocytopenia (ITP) is an acquired disorder characterized by low platelet counts. Steroids are the usual 1^st^-line treatment. Most patients respond with an initial increase in counts but durable remissions are rare and thrombocytopenia often recurs once steroid doses are tapered [1]. Splenectomy, thrombopoietin receptor agonists, and several other treatments have been recommended for 2^nd^ and 3^rd^ line, but experts opinions still differ about the optimal sequence of therapeutic regimens. Treatment decisions are guided by physicians' personal preferences, cost considerations and various regulatory limitations.

At the same time one has to keep in mind that despite the life-threatening character of thrombocytopenia ITP-patients usually do not succumb to their disease [2], [3]. Many live with low platelet counts for years and have remarkably little or even no bleeding symptoms. This is in contrast to other haematologic disorders e.g. lymphoma or leukaemia where a similar degree of thrombocytopenia almost always manifests with bleeding. Chronic ITP is a disease of many years duration and patients have to weigh the potential benefit of treatments against side effects and potential limitations of occupation and daily activities.

Chronic ITP is a rare disorder. Recent studies found a prevalence of 1 in 5.000 [4], [5]. This makes ITP by definition an orphan disease. Few physicians have personal experience in treating large numbers of patients. Patient initiated self support groups have been founded in the US, Britain and other countries. Despite several guidelines published in the last years [6], [7], [8] it is the experience of the authors that many patients are offered treatments that do not follow recommendations, e.g. steroids are prescribed for prolonged periods solely to keep platelet counts in a “safe” range. We therefore concluded that there is a need for more information about the long-term course and experience of ITP-patients. In collaboration with the German ITP-patient support group a questionnaire was developed and made available to the members of the group. Survey items queried patient demographic and clinical characteristics, treatment and side effects and impact on daily functioning.

## Methods

### Procedure

A web-based questionnaire was designed based on clinical literature, expert opinion and patients' experience. After approval from a central institutional review board, in accordance with the Declaration of Helsinki, Good Clinical Practice guidelines, and local laws and regulations survey participants were recruited from the “Giessen ITP Support Group”, a Germany based ITP-patient support group. All participants were invited by mail or e-mail and presented with an online opt-in consent before continuing with the web-based questionnaire. Responses were anonymous to maintain confidentiality.

Eligible study patients had to meet the following criteria:

chronic ITP (defined as having the disease for no less than 12 months),
18 years of age or older.

Patients who did not fulfil the inclusion criteria were not considered for the data analysis.

### The questionnaire

The questionnaire had 45 questions and was divided into three sections:

#### A. Personal and disease history (Q1–13)

Age, gender, duration of disease, platelet count and bleeding history. Patients were asked to report their lowest, highest and “median” (as estimated by the patient) platelet count in the last 12 months. Oral or nasal mucosal bleeds were defined as wet purpura, petechiae and ecchymoses/haematomas as dry purpura. We used these terms because they were familiar to our patients from self support group meetings.

#### B. Treatment history and experience with treatment types (Q14–29)

Corticosteroids, intravenous immunoglobulins, azathioprin, rituximab, splenectomy, etc. including complementary and alternative medicines (CAMs).

#### C. Effect of disease and treatment on daily activities (Q30–45)

Perceived limitations in occupation, recreational activities, (only in women) pregnancy, utilization of medical resources, etc.

Patients were given the option of selecting responses or providing their own response in free form if none of the suggested answers matched their personal experiences.

### Statistics

Descriptive statistics were used to analyze data, Fisher's exact test for comparative testing. Statistical significance was set at a two-sided 5% level. All statistical calculations were performed using standard software (SAS Windows™, version 9.1).

## Results

**Figure 1:**
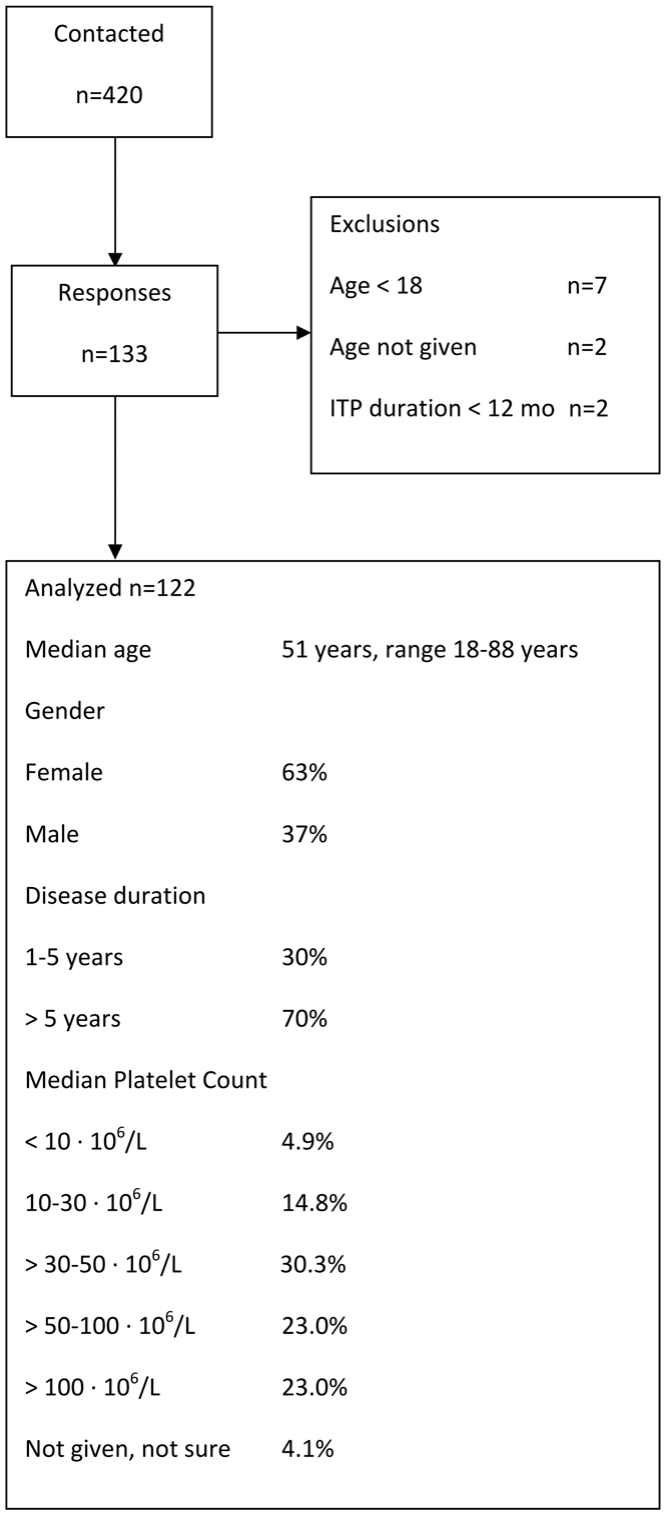
Responses, exclusions and general characteristics of the study population.

Fourhundred twenty patients were contacted by mail or e-mail, 133 responded (32%), 11 had to be excluded, mainly because of age less than 18years. Overall 122 patients were included in the analysis. General characteristics are given in Figure 1.

### A. Personal and Disease History

#### Platelet Count

48% of all patients reported that their lowest platelet count during the preceding 12 months had been ≤30·10^9^/L, with 20% reporting their average platelet count to be ≤30·10^9^/L. The median platelet count below which patients stated they would become concerned was ≤20·10^9^/L.

#### Bleeding History

Fifty patients (41%) reported petechiae or haematomas (dry purpura) and 21 (17%) nasal and oral mucosal bleeds (wet purpura) with a frequency of ‘more than just once or twice’ up to ‘12 times or more during the last year’.

#### Correllation Platelet Count and Symptoms

Out of the twenty-four patients (20%) reporting an average count ≤30·10^9^/L) 17 (71%) reported dry and 5 (21%) wet bleedings ‘more than just once or twice’ up to ‘12 times or more during the last year’. This means vice versa that 7 (29%) of severely thrombocytopenic patients had up to one or two dry bleeds and 19 (79%) up to one or two wet bleeds per year. Among 93 patients with higher counts (average count >30·10^9^/L) these numbers were 32 for dry (34%) and 16 for wet bleeds (17%), respectively (comparison ≤30·10^9^/L vs. >30·10^9^/L p = 0.002 for dry bleeds and p = 0.767 for wet bleeds).

Out of the wenty-eight patients (23%) with an average count >100·10^9^/L during the last year, 6 (21%) reported dry and 2 (7%) wet purpura with a frequency of ‘more than just once or twice’ up to ‘12 times or more during the last year’.

#### Correllation Age and Symptoms

Thirty-six patients (30%) were 60 years or older at the time of the survey. In this subgroup of elderly patients 6 (17%) had an average count ≤30·10^9^/L during the last year with 8 (22%) reporting dry and 4 (11%) wet bleedings ‘more than just once or twice’ or ‘12 times or more during the last year’. For younger patients (<60 years) these numbers were 18 (21%), 41 (48%), and 17 (20%), respectively (comparison elderly vs. young patients p = 0.021 for dry bleeds and p = 0.425 for wet bleeds).

### B. Treatment history and experience with treatment types

#### Hospital Admissions

11% of all patients had been admitted to the hospital during the last 12 months, 3% even more than twice. Of those with an average platelet count of ≤30·10^9^/L, 13% had been admitted to the hospital during the last 12 months and 12% of those with ≥30·10^9^/L (comparison of patients with ≤30·10^9^/L vs. ≥30·10^9^/L p = 1.00). 25% of patients older than 60 years had been admitted to hospital and 6% of those younger than 60 years (comparison elderly vs. young p = 0.005). Of those with ‘more than just one or two’ up to ‘12 or more’ dry bleeds during the last year 10% had been admitted to the hospital during the last 12 months and 13% of those with less bleeds (comparison p = 0.775). For patients with ‘more than just one or two’ up to ‘12 or more’ wet bleeds these numbers were 19% and 10% for those with less wet bleeds (comparison p = 0.269).

#### Therapy with Corticosteroids and other Agents

**Figure 2:**
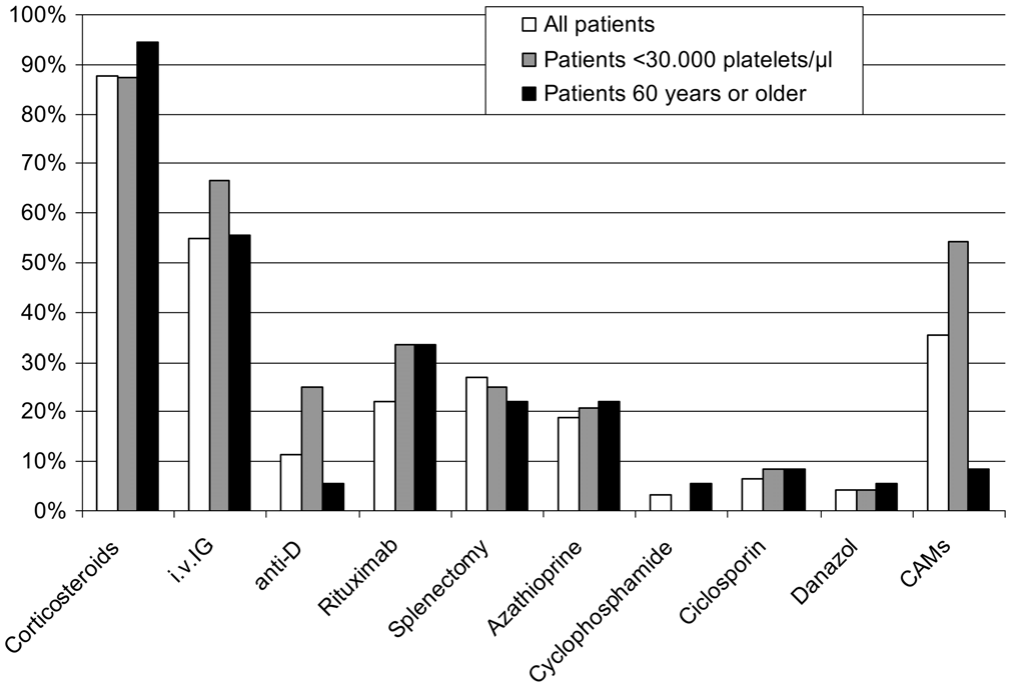
Analysis of the question: ‘What treatments have you ever received for your immune thrombocytopenic purpura?’.

88% of patients stated that they have received corticosteroid therapy at some point during their course of disease. 22% of the patients had been treated with rituximab and 27% were splenectomised. The numbers for those and other treatments are summarized in figure 2. 36% of patients currently use or have used CAMs in the past, among the elderly population this number was much lower with only 8%. We conclude that elderly patients might have less access to the internet and therefore might be less informed about the use of CAMs. Another 7% of patients (n = 9) were treated with “other” treatment options, thereof e.g. platelet (n = 1) and erythrocyte transfusions (n = 1), high dose vitamin C or change in diet.

Thirty patients (28%) were on steroids during the three months prior to the survey, despite a disease duration of 1 year or longer. The most commonly reported severe side effects were weight gain in 48%, moon face in 46%, and personality changes, mood swings in 36%. 69% stated they would take steroids only if there was absolutely no other option and 17% would not take steroids any more at all.

27 patients had been treated with rituximab. 7% reported severe infusion reactions and 52% stated they would try to avoid or even refuse rituximab in the future. 33 patients had been splenectomised, 58% of which would advise other patients against this procedure. Following splenectomy, 42% of the patients had to receive subsequent treatment with rituximab, IVIGs, etc.

### C. Effect of disease and treatment on daily activities and pregnancies

11% of the patients felt ‘often’ or even ‘extremely’ affected by their disease at school or occupational activities, 7% at daily non-occupational activities. 13% of those patients with an average platelet count of <30·10^9^/L and 12% of those with an average count ≥30·10^9^/L felt ‘often’ or ‘extremely’ limited at school/occupational activities, 4% and 9% during daily non-occupational activities, respectively. Statistical analysis did not show any significant differences for patients with platelet counts <30·10^9^/L vs. ≥30·10^9^/L (comparison of impairment of occupational/school activities p = 1.000, impairment of non-occupational activities p = 0.68). 20% stated that they experienced problems with health care coverage. 57% consulted a haematologist for their disease while 16% saw a specialist for alternative medicine or did not seek medical attendance at all.

50 women responded to questions concerning pregnancy. 14 (28%) stated that they had been advised not to become pregnant. 23 (19%) reported pregnancies and 10 (44%) were treated for their ITP during pregnancy. 2 (9%) of the newborns had been thrombocytopenic.

## Discussion

ITP is an uncommon disorder. A prevalence of less than 1 in 5.000 [4] qualifies ITP as an orphan disease. Except for 1^st^-line therapy with corticosteroids recommended by most national and international guidelines there is no generally accepted treatment consensus for 2^nd^- and 3^rd^-line treatment. There are few reports about the long-term clinical course of chronic ITP except for splenectomised patients [9], [10] but splenectomised patients are a minority among chronic ITP-patients. Only very recently patients' perspective on treatment related side effects and quality of life has moved into the focus of clinical research and guideline development [11], [12], [13]. Our survey reveals

that few patients with chronic ITP have severe bleeding complications,
that the hospital admission rate is not higher in ITP patients with low platelet counts compared to higher platelet counts and not higher in patients with frequent wet or dry bleeds compared to those with rare or absent bleeds,
that the hospital admission rate is higher in elderly patients with ITP,
that most patients have been treated with corticosteroids and many still are even after one year,
that side effects from corticosteroids are perceived as particularly bothersome,
that only a minority of patients with chronic ITP undergo splenectomy,
that many patients receive “off-label” therapies (e.g. rituximab, cyclosporine, danazol),
that even more than one third of patients uses complementary and alternative medicines (CAMs).

In 1996, the ASH practice-guideline stated that “withholding treatment was inappropriate for patients with a platelet count ≤20·10^9^/L, regardless of their symptoms” [7]. This same attitude prevailed in the 2003 UK recommendations with a slightly higher threshold of 30·10^9^/L [6]. Both recommendations have lead physicians to base treatment decisions solely on platelet counts. The new international consensus report does not dismiss this paradigm but notes that asymptomatic patients with platelets >50·10^9^/L rarely need therapy [8]. All these thresholds have been arbitrarily defined based on expert opinion. Two retrospective studies describe a correlation of bleeding events with patient age >60 years and platelet counts <30·10^9^/L [14], [15]. However, none of these studies records whether patients had severe bleedings before and how their bleeding history predicts future events. One of the studies also includes non-ITP-patients [15].

We assume that the number of hospital admissions might serve as a surrogate marker for severe bleeding events (because those are usually not treated as outpatients). Furthermore we observed that patients with a low platelet count (<30·10^9^/L) have more dry but not more wet bleedings than patients with higher counts. The reason for this difference is not clear. However, patients with low counts do not have more hospital admissions than patients with higher counts. Elderly patients are admitted more frequently but this may have other reasons and need not be bleeding-related. Together, these observations suggest that platelet counts correlate with minor bleeding events (dry bleeds) but not with severe bleedings.

Patients receive steroids for prolonged periods of time or they are subjected to splenectomy solely to keep platelets above a presumably safe threshold. A study in children shows that therapy of low platelet counts had no effect on the subsequent development of severe bleedings [16]. On the other hand several recent studies in adults with chronic ITP find less severe bleeding when platelet counts rise above 50·10^9^/L [17], [18]. However, these studies do not address the question whether thrombocytopenic ITP-patients with no or only minor bleeding benefit clinically from raising their counts. The results of this survey support the recent German ITP guideline recommendations to base treatment primarily on the severity of clinical symptoms [19]. Khellaf et al. recommend to base treatment decisions not on platelet count but clinical bleeding signs [20]. Psaila et al. also find that intracranial haemorrhage in children is not predicted by platelet count [21].

The new international consensus report recommends to grade bleeding severity by counting petechiae and measuring the size of haematomas [8], [22]. Surprisingly among 28 patients who reported their medium platelet count during a 12 month period to be over 100·10^9^/L 6 (22%) respectively 2 (7%) stated they had dry and wet purpura more than just once or twice a month. This might indicate that treatment decisions should not be based on patient reports of petechiae and haematomas, because the prognostic relevance is not yet clear.

Almost all patients in this study had at some point received corticosteroids, 28% were still taking steroids during the three months before the survey (despite disease duration >1 year). 69% stated that they would avoid steroids whenever possible, 17% would not take them any more at all. The adverse effects most often reported by patients were weight gain, moon face and mood swings. This is in accordance with recent studies on quality of life in ITP-patients [11], [23], [24].

As mentioned above the major complaints during steroid treatment were weight gain, moon face and personality changes. At the same time only 12% of patients were bothered by severe gastric symptoms, 9% reported severe hypertension, and 3% severe hyperglycemias. This contrasts with physicians' perception of steroid side effects. Patients are usually informed about the risks of ulcers, hypertension, infections and diabetes. Weight gain is generally not considered a contraindication by physicians. There has been a similar observation in cancer patients who rank side effects differently than their physicians and who are often dissatisfied with the handling of treatment-related toxicities [25].

Of notice, despite guideline recommendation only 27% of patients had been splenectomised. On the other hand 44% of patients received “off-label” drugs (22% rituximab, 11% anti-D, 7% ciclosporin, 4% danazol), when only the following drugs were approved in Germany for the treatment of immune thrombocytopenia: steroids, immunoglobulins, azathioprine, thrombopoietin receptor agonists and vinca alkaloids. The rate of splenectomies is low, not only in Germany, but also in other countries [26], [27]. This observation suggests that patients are willing to accept even unlicensed therapies to avoid surgery. The recently introduced term “splenectomy-sparing therapy” indicates that avoidance of splenectomy has become a valuable therapeutic goal [28]. German public health insurance usually does not cover cost for unlicensed therapies. A previous survey conducted 5 years earlier had shown that almost 40% of patients had the feeling that financial capabilities affected treatment recommendations [29]. In this study still 20% report problems with cost coverage. Most recently, thrombopoietin receptor agonists have been denied approval in Europe for 2^nd^-line therapy in patients without splenectomy (except for when splenectomy is contraindicated) despite phase III data showing efficacy. All this could add to the frustration of patients and partially explains the frequent use of CAMs, which are widely used after corticosteroids, more often than any other evidence-based treatment.

Severe maternal or neonatal bleedings are rare when pregnant women with chronic ITP are managed by a multiprofessional team [30]. In this study were 77 women, of whom 50 responded to questions concerning pregnancy and ITP. Many reported that they had been advised not to become pregnant. 10 of 23 women (43%) received treatment for thrombocytopenia during pregnancy. Two of the newborns were thrombocytopenic. There is an urgent need for clinical centres with gynaecologists and haematologists experienced in the treatment of chronic ITP and providing 24 hour service to pregnant women with ITP.

Our study has several limitations. All participants were recruited from the register of an ITP self support group and retrospectively reported on their course of disease. Patients in self support groups have usually received - and failed - several lines of therapy and are therefore more critical to the efficacy and side effects of therapies. We also cannot exclude that the web-based format of this survey has deterred some elderly or less capable patients. However, the percentage of Germans using the internet is constantly growing, with the highest growth rates in the elderly population. With regards to age and gender this survey seems comparable to other publications [4], [27]. The members of the Giessen ITP support-group are usually mobile and able to attend the annual group meeting. We assume that this study has a pre-selection bias because patients with more severe, debilitating or lethal bleedings might be underrepresented. It was not the purpose of this study to give a representative picture of all chronic ITP-patients in Germany, but rather from a large and clinically relevant group (to our knowledge this is the only German ITP support group with a considerable number of members (n∼500)). Even if some patients with severe disease were not included this does not devaluate the experiences of the majority of patients.

In the results section we report patients' statements about the frequency of dry and wet bleedings. We are aware that it is difficult for patients to identify dry and wet bleedings.

In conclusion, this study reveals that most chronic ITP-patients have a benign clinical course. Low platelet count and frequency of wet and dry purpura do not predict a higher risk of hospital admissions, but age does. Glucocorticoids are still the most common therapy for chronic ITP and often used for prolonged periods. Complementary and alternative treatments already come second and less than ⅓ of the patients are splenectomised. Many patients receive off-label treatments. The frequent use of complementary medicines suggests dissatisfaction with conventional health care. There is a major need for adequate counselling and care for pregnant ITP-patients.
